# B-Cell Lymphopoiesis Is Regulated by Cathepsin L

**DOI:** 10.1371/journal.pone.0061347

**Published:** 2013-04-09

**Authors:** Maria Noel Badano, Gabriela Lorena Camicia, Gabriela Lombardi, Andrea Maglioco, Gabriel Cabrera, Hector Costa, Roberto Pablo Meiss, Isabel Piazzon, Irene Nepomnaschy

**Affiliations:** 1 Laboratorio de Inmunología Experimental, Instituto de Medicina Experimental-Consejo Nacional de Investigaciones Científicas y Técnicas, Academia Nacional de Medicina, Buenos Aires, Argentina; 2 Laboratorio de Inmunofarmacología Tumoral, Centro de Estudios Farmacológicos y Botánicos, Consejo Nacional de Investigaciones Científicas y Técnicas, Facultad de Medicina, Universidad de Buenos Aires, Buenos Aires, Argentina; 3 Centro de Estudios Oncológicos, Academia Nacional de Medicina, Buenos Aires, Argentina; Istituto Superiore di Sanità, Italy

## Abstract

Cathepsin L (CTSL) is a ubiquitously expressed lysosomal cysteine peptidase with diverse and highly specific functions. The involvement of CTSL in thymic CD4+ T-cell positive selection has been well documented. Using CTSL^nkt/nkt^ mice that lack CTSL activity, we have previously demonstrated that the absence of CTSL activity affects the homeostasis of the T-cell pool by decreasing CD4+ cell thymic production and increasing CD8+ thymocyte production. Herein we investigated the influence of CTSL activity on the homeostasis of peripheral B-cell populations and bone marrow (BM) B-cell maturation. B-cell numbers were increased in lymph nodes (LN), spleen and blood from CTSL*^nkt/nkt^* mice. Increases in splenic B-cell numbers were restricted to transitional T1 and T2 cells and to the marginal zone (MZ) cell subpopulation. No alterations in the proliferative or apoptosis levels were detected in peripheral B-cell populations from CTSL*^nkt/nkt^* mice. In the BM, the percentage and the absolute number of pre-pro-B, pro-B, pre-B, immature and mature B cells were not altered. However, *in vitro* and *in vivo* experiments showed that BM B-cell production was markedly increased in CTSL*^nkt/nkt^* mice. Besides, BM B-cell emigration to the spleen was increased in CTSL*^nkt/nkt^* mice. Colony-forming unit pre-B (CFU pre-B) assays in the presence of BM stromal cells (SC) and reciprocal BM chimeras revealed that both BM B-cell precursors and SC would contribute to sustain the increased B-cell hematopoiesis in CTSL*^nkt/nkt^* mice. Overall, our data clearly demonstrate that CTSL negatively regulates BM B-cell production and output therefore influencing the homeostasis of peripheral B cells.

## Introduction

B-cell development occurs continuously during life. In adult mice, this process is initiated in the bone marrow (BM) where hematopoietic stem cells differentiate through a series of intermediate stages during which cells are thought to become progressively more restricted in their developmental potential. Once the B-lineage restricted stage is reached, B-cell progenitors execute a programmed development, first rearranging the immunoglobulin heavy chain gene at the pro-B stage, then undergoing multiple rounds of clonal expansion at the pre-B stage and finally rearranging the light chain gene to yield newly formed B cells expressing surface IgM. These immature B cells are exported primarily to the spleen where they progress through stages of immature transitional B cells and develop into mature naïve B cells [1].

Cathepsin L (CTSL) is an abundant and ubiquitously expressed lysosomal cysteine peptidase which degrades a wide range of cytoplasmic and nuclear proteins [2]. On the other hand, about 10% of CTSL is physiologically secreted and can be extracellularly activated [3]. There, it is capable of processing extracellular matrix (ECM) proteins such as fibronectin, laminin, elastin and diverse type of collagens [3]–[5]. A considerable body of evidence has accumulated in the last years showing the involvement of CTSL in diverse and highly specific functions such as epidermal homeostasis and regulation of the hair cycle [6]–[9], maintenance of the heart structure and function [10]–[12], endothelial progenitor cell-induced neovascularization [13] and processing of proneuropeptides into peptide neurotransmitters and hormones [14], [15]. A role for CTSL in the development and progression of cancer has also been reported [16], [17].

Several cathepsins contributed in the processing of both antigens and self-antigens to antigenic peptides [18]–[20]. Regarding the thymic compartment, it has been demonstrated that CTSL plays an important role in the MHC class II-mediated peptide presentation in thymic epithelial cells, acting both in the invariant chain degradation [21] and in the generation of MHC class II-bound peptide ligands presented by cortical thymic epithelial cells [18]. Consequently, CTSL KO mice exhibit a marked reduction in the percentage of CD4+ cells in the thymus and spleen. We and others have shown [22]–[24] that CTSL*^nkt/nkt^* mice -which carry an inactivating mutation in the *Ctsl* gene [24]- also have an early impairment during positive selection of CD4+ thymocytes.

Lymph nodes (LN) from CTSL*^nkt/nkt^* mice are enlarged and show an increased number of lymphocytes. In spite of the low rate of CD4+ cell thymic production, the number of LN CD4+ T cells is similar to that of wild-type (wt) mice due to a marked increase in their proliferative level. In addition, the number of LN CD8+ cells is significantly increased correlating with an increased thymic export of CD8+ cells [25].

Recently, a role for cathepsin B in B cell development has been proposed [26].However, despite the progress made in elucidating the role of CTSL in CD4 and CD8 T cell homeostasis, the influence of CTSL on B cells has not yet been addressed. Thus, the aim of this work was to investigate whether CTSL activity affects the B-cell compartment.

## Materials and Methods

### Mice

The following specific pathogen-free mice were used: BALB/c.Cg-Ctsl*^nkt^* (CTSL*^nkt/nkt^*), BALB/c and CByJ.B6-Tg (UBC-GFP) 30Scha/J transgenic BALB/c expressing enhanced green fluorescent protein (GFP) under the direction of the human ubiquitin C promoter (BALB/c.GFP). The origin and development of the CTSL*^nkt/nkt^* congenic (N>12) strain has been previously described [24], [25]. CTSL*^nkt/nkt^* mice were identified by their alopecy and by the presence of a deletion in both copies of the *Ctsl* gene. The deletion was detected by RT-PCR (sense primer 5′CAATCAGGGCTGTAACGGAGG 3′, antisense primer 5′CATTGAGGATCCAAGTCATG3′) as previously described [25]. BALB/c.GFP mice were purchased from the Jackson Laboratories, Bar Harbor, Maine. These mice express GFP in all tissues examined including those of hematopoietic origin.

Housing and breading in our animal facility (IMEX-CONICET, Academia Nacional de Medicina) and all experimental procedures were carried out according to the policies of the Academia Nacional de Medicina, based on “Guide for Care and Use of Laboratory Animals. Bethesda, MD: National Institutes of Health; 1985. NIH publication N.85-23”. Experiments were approved by the ethical committee of the IMEX-CONICET (Permit number 1009).

### Cell suspensions

LN cell suspensions were prepared from a pool of axillary and inguinal LN as previously described [25]. This pool of LN was used to calculate the number of cells per LN. To obtain BM cells, femurs were dissected free from the muscle, cut both at the knee and hip, and removed. BM cell suspensions were prepared by repeatedly flushing the cells from femurs using a 23-gauge needle with DMEM (GIBCO by Invitrogen, CA, USA), containing 5% FBS (GIBCO) [27]. Mononuclear cells from BM, spleen and blood were isolated by centrifugation on Ficoll-Triyoson gradients.

### Antibodies

Anti-murine mAb directly conjugated to FITC, PE, Cy, TruRed or APC were used for fluorescence-activated cell sorting. FITC-labeled mAb included anti-HSA (M1/69), anti-IgM (R6-60.2), anti-IgD (11-26c.2a), and anti-B220 (RA3-6B2). PE-labeled antibodies included anti-CD19 (1D3), anti-CD43 (S7), anti-B220, anti-CD23 (B3B4) and anti-IgM. Cy-labeled anti-B220, TruRed-labeled anti-IgD and APC-labeled anti-CD21 (7G6) mAb were used. All antibodies were purchased from BD Pharmingen (San Diego, CA, USA) except for TrueRed anti-IgD which was purchased from Biolegend (San Diego, CA, USA).

### Flow cytometric staining

Cells (1×10^6^) from BM, LN or spleen resuspended in RPMI 1640 without phenol red (GIBCO) containing 3% FBS, 0.1% sodium azide, and 10 mM HEPES (GIBCO), were incubated in one step with the appropriate mAb [28]. Acquisition of 20–50.000 cells was performed using a FACScan or a dual-laser FACSAria flow cytometer (BD Biosciences). Dead cells were excluded on the basis of forward and side scatter. Background values obtained with fluorochrome conjugate isotype controls (BD Pharmingen) were subtracted. Results were analyzed using CellQuest software (BD Immunocytometry Systems).

### Bromodeoxyuridine (BrdU) pulse labeling

Pulse labeling was achieved by injecting mice i.p. with 2 mg of BrdU (Sigma-Aldrich Corp, St. Louis, MO, USA) for 2 days. Additional mice injected with sterile PBS were used to determine background values. Cells from BrdU-treated mice were stained for surface expression of IgM, IgD, CD43 and B220, washed, fixed and permeabilized using BD Cytofix/Cytoperm Buffer. Then cells were washed, treated with 30 µg of DNase I (Sigma), washed and stained with anti-BrdU-FITC before FACS analysis [29].

### Apoptosis assays

Propidium iodide (PI) (Sigma) or Annexin V (BD Pharmingen) were independently used to detect apoptotic B cells. For PI staining, cells resuspended in PBS containing 3% FBS were incubated with FITC-labeled mAb directed against surface markers. Cells were washed, fixed with 1 ml of ethanol 70% and stored at 4°C for 24 h. Then, cells were washed and resuspended in PBS containing 1% glucose, 1 mg/ml RNase A (Sigma), and 20 µg/ml PI. After 30 min of incubation, cells were acquired on a FACScan flow cytometer [25]. For Annexin V staining, cells were incubated with the appropriate surface mAb, washed with PBS, resuspended in 150 µl of Annexin V binding buffer and stained with 1 µl of Annexin V. After 30 min of incubation cells were analyzed by FACS.

### Cell cycle analysis

For DNA content analysis, PI or 7-amino-actinomycin-D (7-AAD) (BD Pharmingen) [30] were used. PI staining was performed as described above [25]. For 7-AAD staining [29], cells were stained for the appropriate surface markers, washed, fixed and permeabilized using BD Cytofix/Cytoperm Buffer. Cells were washed, treated with 30 µg of DNase I, washed and stained with 20 µl of 7-AAD before FACS analysis.

### B cell isolation

B220+ cells from LN were purified by positive selection by using CD45R (B220) MicroBeads (Miltenyi Biotec, Germany) according to the manufacturer's protocol. The B cell purity (%CD19+cells, >95%) was determined by flow cytometry. Cytocentrifuge slides were prepared by centrifugation of samples at 80 *g* for 5 min (Cytospin 4; Shandon, Pittsburg,USA) and stained with May-Grunwald Giemsa.

### BM autoreconstitution assays

Wt and CTSL*^nkt/nkt^* mice were exposed to 500 rad of ionizing radiation and BM and spleen B-cell subpopulations were analyzed by FACS thirteen days post-irradiation [31]. Mice were given drinking water supplemented with 2 mg/ml neomycin sulfate (Sigma-Aldrich) 2 days before and following irradiation.

### Colony-forming unit pre-B (CFU pre-B) assay

Femoral BM cells (1×10^5^) were cultured in 35-mm^2^ plastic Petri dishes containing 1 ml of the recombinant IL-7 (rIL-7)-supplemented MethoCult M3630 medium (StemCell Technologies, Vancouver, Canada). Colonies containing more than 30 cells were scored under an inverted microscope after culture for 7 days at 37°C in a fully humidified atmosphere with 5% CO_2_ [32].

### BM stromal cells (SC) cultures

BM cells were cultured in 24-well culture plates in DMEM supplemented with 20% FBS, 0.1% 2-mercaptoethanol (ME), 1% L-glutamine, at 37°C in a humidified atmosphere at 5% CO_2_ for 24 hours. Thereafter, nonadherent cells were washed out and the remaining cells were further cultured under the same conditions [32].

### Co-culture of BM stromal monolayers and pro-B cell-rich populations

To obtain a pro-B cell-rich population a bulk culture of pooled BM cells stimulated with rIL-7 was performed as described by Tsuboi el al [32]. Briefly, BM cells from wt and CTSL*^nkt/nkt^* mice were cultured at 1×10^6^ cells/ml in DMEM supplemented with 20% FBS, 2×10^−5^ *M* 2-ME, 1% L-glutamine, and 2 ng/ml of murine rIL-7 (R&D Systems) and plated in six-well culture trays. Nonadherent cells were harvested after 4 days of culture. This bulk culture provided a highly rich source of IL-7-responsive pro-B cells (>70% B220^lo^CD43^+^HSA^hi^). Pro-B cell-rich populations were suspended at 5×10^5^/ml in DMEM supplemented with 20% FBS, 0.1% ME, 1% L-glutamine and 1 ng/ml of murine rIL-7. Aliquots (1.0 ml) of this cell suspension were added to established SC monolayers in 24-well flat-bottomed trays and co-cultured at 37°C in a fully humidified atmosphere of 5% CO_2_. Nonadherent cells were harvested after 3 days, counted, and cloned using the CFU-pre-B colony assay system.

### Reciprocal BM chimeras

Reciprocal BM chimeras were constructed as described by Mollenn et al. [33] with minor modifications. Briefly, 2×10^6^ BM cells from wt, CTSL*^nkt/nkt^* were i.v. injected into 8-week old mice that had received 900 rad of whole body irradiation 24 h previously. Parallel experiments using BALB/c.GFP instead of wt mice were developed in order to confirm the B-cell donor-origin in reconstituted mice. 2 days before and following irradiation, mice were given drinking water supplemented with 2 mg/ml neomycin sulfate. Twelve days after adoptive transfer, BM and spleen cells were harvested from recipient mice for analysis.

### Statistical analysis

The two-tailed Student´s *t* test was used to assess the statistical significance of the results. Results are expressed as (mean ± SD). A value of *p*<0.05 was considered indicative of a significant difference. Except where specified, the figures and tables contain representative results from experiments repeated independently 3 times.

## Results

### The number of B cells is increased in LN from CTSL*^nkt/nkt^* mice

**Figure 1:**
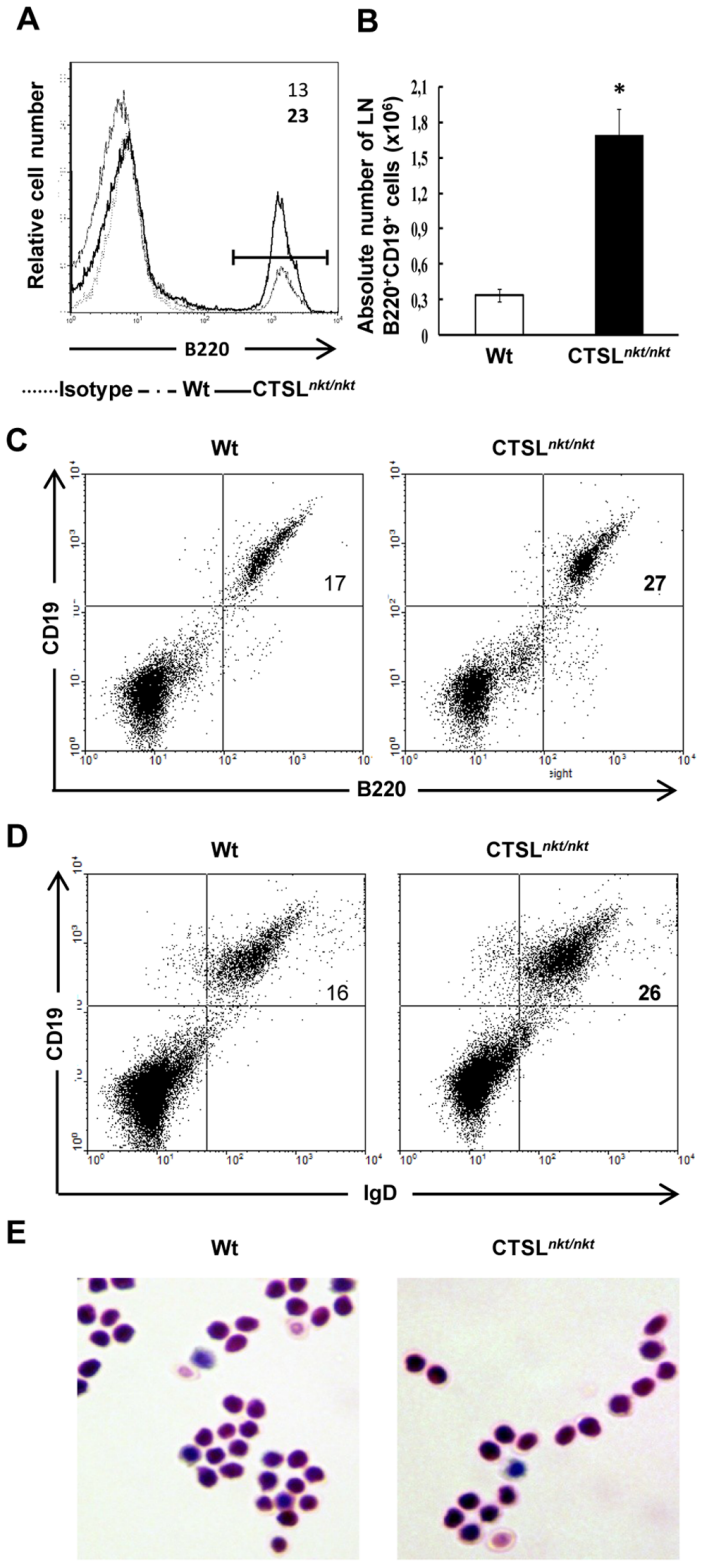
LN B-cells are increased in CTSL*^nkt/nkt^* mice. LN cells from wt and CTSL*^nkt/nkt^* mice were stained with anti-B220, CD19 and IgM and analyzed by FACS. (A) Representative histograms of LN B220^+^ cells are shown. (B) Absolute numbers (mean ± SD; n = 4) of LN B220+CD19+cells. (C) Representative dot plots of CD19+B220+LN cells (D) Representative dot plots of CD19+IgD+LN cells. E. Purified B220+LN cells spotted on a cytospin slide. In both, Wt and CTSL nkt/nkt animals, similar groups of mainly small lymphoid cells are seen with round to oval eccentric nuclei, coarse chromatin, and scanty homogeneous cytoplasm. May-Grunwald Giemsa staining (original magnification x1000). *p<0.001 compared to wt. Experiments were performed four times with similar results.

We first investigated whether CTSL*^nkt/nkt^* mice showed alterations in the number of B cells in the enlarged LN of these mice. As can be observed in F1g 1A, the percentage of B220+cells was significantly increased in LN of CTSL*^nkt/nkt^* mice. Both the percentage and absolute number of B220+CD19+LN cells were markedly increased in CTSL*^nkt/nkt^* mice (Fig 1B–C). These data indicate that the number of LN B cells is markedly increased in mutant mice. LN B cells from mutant and wt mice did not show significant differences in the level of expression of B220 (Fig 1A and [34]). All LN B cells were IgM^lo^ IgD^hi^ whereas IgM^hi^ IgD^+/hi^ cells were not detected (data not shown) indicating that LN B cells of CTSL*^nkt/nkt^* mice are phenotypically mature. No significant morphological differences were detected between B cells from wt and mutant mice (Fig 1 E).

PI staining revealed no alterations in B-cell basal proliferative levels in LN from CTSL*^nkt/nkt^* mice (percentage of S-G2/M cells within B220+cells: 1.1±0.3 *vs* 1.6±0.7, in wt *vs* CTSL*^nkt/nkt^* mice, mean + SD, n = 4). Neither B-cell basal apoptosis levels were modified as measured by PI staining (percentage of hypodiploid cells within B220+ cells: 0.32±0.06 *vs* 0.28±0.04, in wt *vs* CTSL*^nkt/nkt^* mice, mean + SD, n = 4), thus indicating that alterations in proliferation or apoptosis levels are not involved in the increase in the number of LN B cells in mutant mice.

### The number of transitional B cells is increased in the spleen and peripheral blood of CTSL*^nkt/nkt^* mice

**Figure 2:**
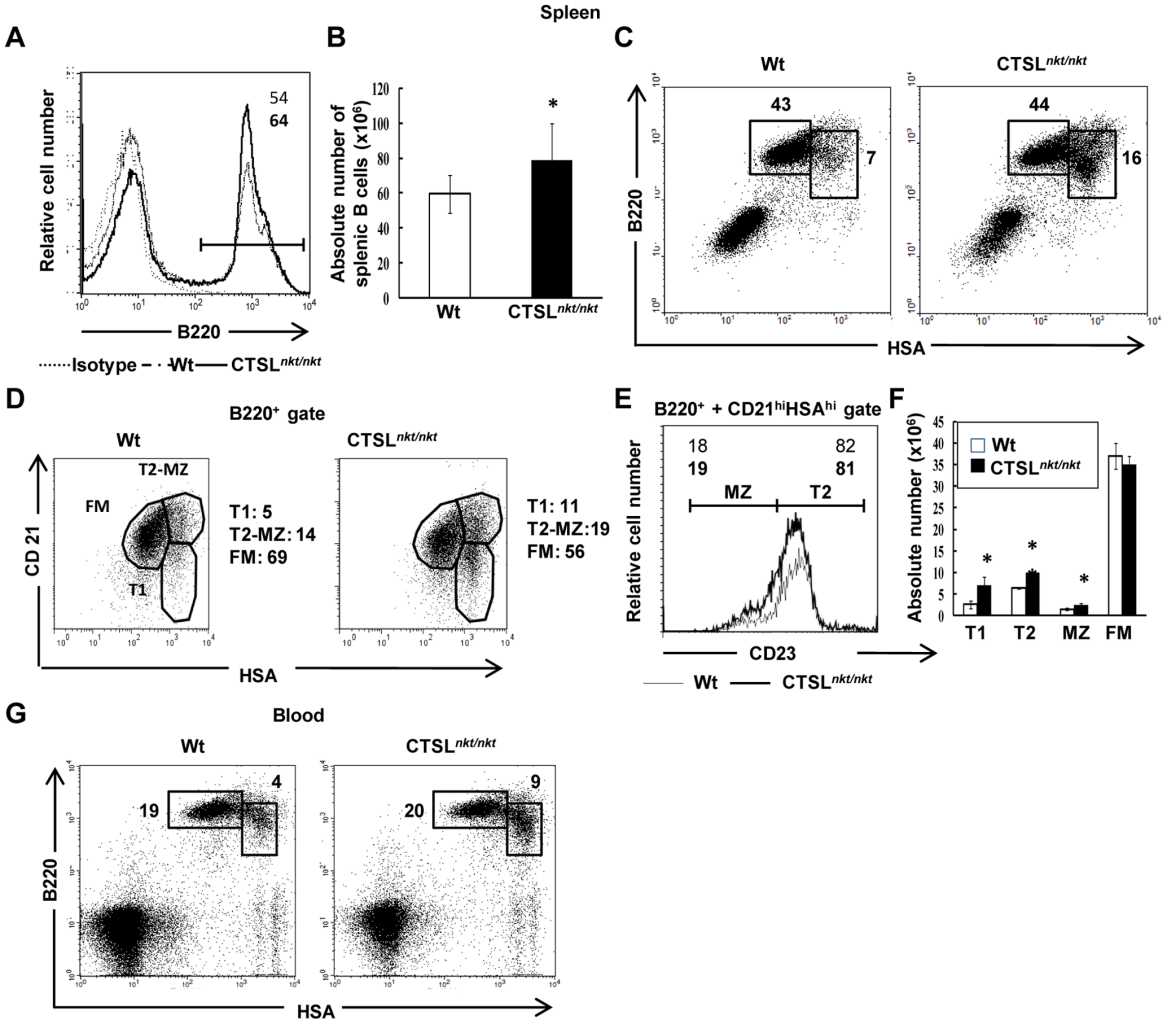
Splenic and blood transitional B-cells numbers are increased in CTSL*^nkt/nkt^* mice. Splenic (A–F) and blood (G) mononuclear cells from wt and CTSL*^nkt/nkt^* mice were stained with anti-B220, anti-HSA, anti-CD21 and anti-CD23 before analysis by FACS. (A) Representative histograms of splenic B220^+^ cells are shown. (B) Absolute numbers (mean ± SD; n = 4) of splenic B cells. (C) Representative dot plots of splenic transitional (HSA^hi^B220^lo/+^) and mature (HSA^lo^B220^hi^) B cells are shown. Percentages of transitional and mature B cells are indicated. (D) Representative dot plots of splenic T1 (CD21^lo^HSA^hi^), T2-MZ (CD21^hi^HSA^hi^) and FM (CD21^int^HSA^lo^) B cells. Percentages of each subset within B220^+^ cells are indicated. (E) Representative histograms of CD23 surface expression within the splenic B220^+^CD21^hi^HSA^hi^ population. Percentages of T2 and MZ B cells are indicated. (F) Absolute numbers (mean ± SD; n = 4) of splenic T1, T2, MZ and FM B cells. (G) Representative dot plots of blood transitional (HSA^hi^B220^lo/+^) and mature (HSA^lo^B220^hi^) B cells are shown. Percentages of transitional and mature B cells are indicated. *p<0.05; **p<0.0005 compared to wt.

In the spleen, the total number of lymphocytes was not found to be altered in mutant mice [25]. However, both the percentage and the absolute number of splenic B cells were slightly although significantly increased (Fig 2A–B). Splenic B lymphocytes comprise mature and transitional B cells that arrive from the BM to complete their maturation. Transitional B cells are divided into T1 and T2, while the mature B-cell compartment involves the follicular mature (FM) and the marginal zone (MZ) cells [35]. As can be observed in Fig 2C, FACS analysis showed that mutant mice have a higher percentage of transitional B cells (HSA^hi^B220^lo/+^). Based on HSA and CD21 expression, the analysis of B splenic subsets revealed that the percentages of T1 (CD21^lo^HSA^hi^) and T2+MZ (CD21^hi^HSA^hi^) B cells were increased in CTSL*^nkt/nkt^* mice as compared to wt mice (Fig 2D). In contrast, the percentage of FM (CD21^int^HSA^lo^) B cells was decreased in mutant mice. Analysis of CD23 expression in CD21^hi^HSA^hi^ cells (T2+MZ) showed that the proportion of T2 and MZ B cells was similar in wt and CTSL*^nkt/nkt^* mice, indicating that both subsets were equally increased in mutant mice (Fig 2E). The absolute numbers of T1, T2 and MZ B cells were significantly increased in CTSL*^nkt/nkt^* mice, whereas the number of FM B cells was normal (Fig 2F).

Incorporation of 7-AAD was used to measure the proliferation [30] of B splenocytes. Transitional and mature B splenocytes showed similar proliferative levels in mutant and wt mice (percentage of S-G2/M cells within HSA^hi^B220^lo/+^ cells: 1.6±0.5 *vs* 1.4±0.4; percentage of S-G2/M cells within HSA^lo^B220^hi^ cells: 0.18±0.05 *vs* 0.14±0.05, in wt *vs* CTSL*^nkt/nkt^* mice, mean ±SD, n = 4). In addition, no differences were observed in the apoptosis levels (percentage of Annexin V+ cells within HSA^hi^B220l^o/+^ cells: 25.3±4 .0 *vs* 29.7±3.1; percentage of Annexin V+ cells within HSA^lo^B220^hi^ cells: 15±3 *vs* 14±1, in wt *vs* CTSL*^nkt/nkt^* mice, mean ± SD, n = 4).

Regarding peripheral blood, B-cell levels were also increased in CTSL*^nkt/nkt^* mice (Fig 2G). As in the spleen, this increase was restricted to transitional B cells (HSA^hi^B220^lo/+^) and involved both transitional T1 (IgM^hi^IgD^low^) and T2 (IgM^hi^IgD^hi^) B-cell stages (absolute numbers of T1 cells: 0.74±0.13×10^5^ *vs* 1.80±0.20×10^5^, in wt *vs* CTSL*^nkt/nkt^* mice, mean±SD, n = 4, p<0.005, absolute numbers of T2 cells: 0.60±0.11×10^5^ *vs* 1.31±0.10×10^5^, in wt *vs* CTSL*^nkt/nkt^* mice, mean ± SD, n = 4, p<0.001). Regarding the mature B-cell population (IgM^lo^IgD^hi^), no differences in the absolute numbers were detected between normal and mutant mice (absolute numbers of mature B cells: 4.59±1.25×10^5^ *vs* 4.70±0.95×10^5^, in wt *vs* CTSL*^nkt/nkt^* mice, mean ±SD, n = 4).

Thus, both in the spleen and peripheral blood of CTSL*^nkt/nkt^* mice, the number of transitional B cells is increased.

### The entry of newly formed B cells into the spleen is increased in CTSL*^nkt/nkt^* mice

**Figure 3:**
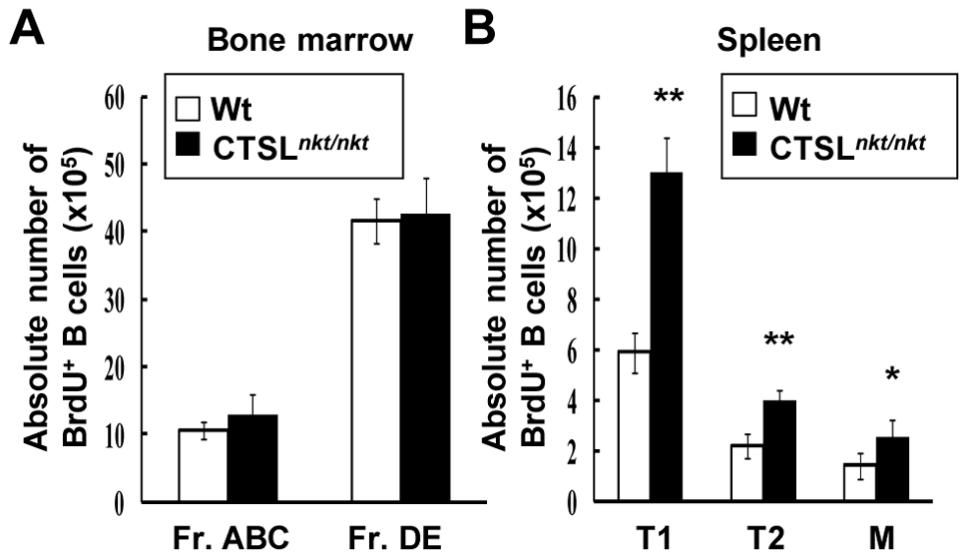
The entry of newly formed B cells into the spleen is increased in CTSL*^nkt/nkt^* mice. BM and splenic cells from mice injected i.p. with BrdU for 2 days were stained with anti-B220, anti-CD43, anti-IgD and anti-IgM before FACS analysis. (A) Proliferation of BM B-cell precursors classified according to Hardy^41^: Fractions ABC (B220^lo^CD43^+^) and Fractions DE (B220^lo^CD43^−^). Absolute numbers (mean ± SD; n = 4) of BrdU^+^ cells within fractions ABC and DE are shown. (B) Emigration of newly formed BM B-cells to the spleen. Splenic BrdU^+^ B cells represent B-cell precursors that have recently left the BM. Absolute numbers (mean ± SD; n = 4) of BrdU+ cells within splenic T1 (IgM^hi^IgD^low^), T2 (IgM^hi^IgD^hi^) and M (IgM^low^IgD^hi^) B cells are shown. *p<0.05; **p<0.001 compared to wt. Representative results from one of three independent experiments are shown.

The fact that splenic transitional B cells were increased in the absence of alterations in their proliferative and apoptosis levels led us to investigate whether BM B-cell emigration to the spleen was increased in CTSL*^nkt/nkt^* mice. Taking into account that BM pro-B and pre-B cells proliferate actively [36] and that the vast majority of transitional B splenocytes are in the G0/G1 phase of the cell cycle [37], [38] (and our own results), a 2 day-BrdU pulse was used as a cohort labeling tool to follow the post-mitotic differentiation of recently labeled pro-B and pre-B cells [39], [40]. Since splenic B cells derive from BM proliferating cells, the BrdU+ splenic B cells would mostly represent BM immature B cells that have recently emigrated to the spleen. As can be observed in Fig 3, whereas no changes in the number of BM BrdU^+^ B cells were detected, the absolute number of BrdU-labeled transitional and mature B cells was increased in the spleen of CTSL*^nkt/nkt^* mice, indicating that the entry of newly formed B cells into the spleen is increased in these mice.

### BM B-cell production is increased in CTSL*^nkt/nkt^* mice

**Figure 4:**
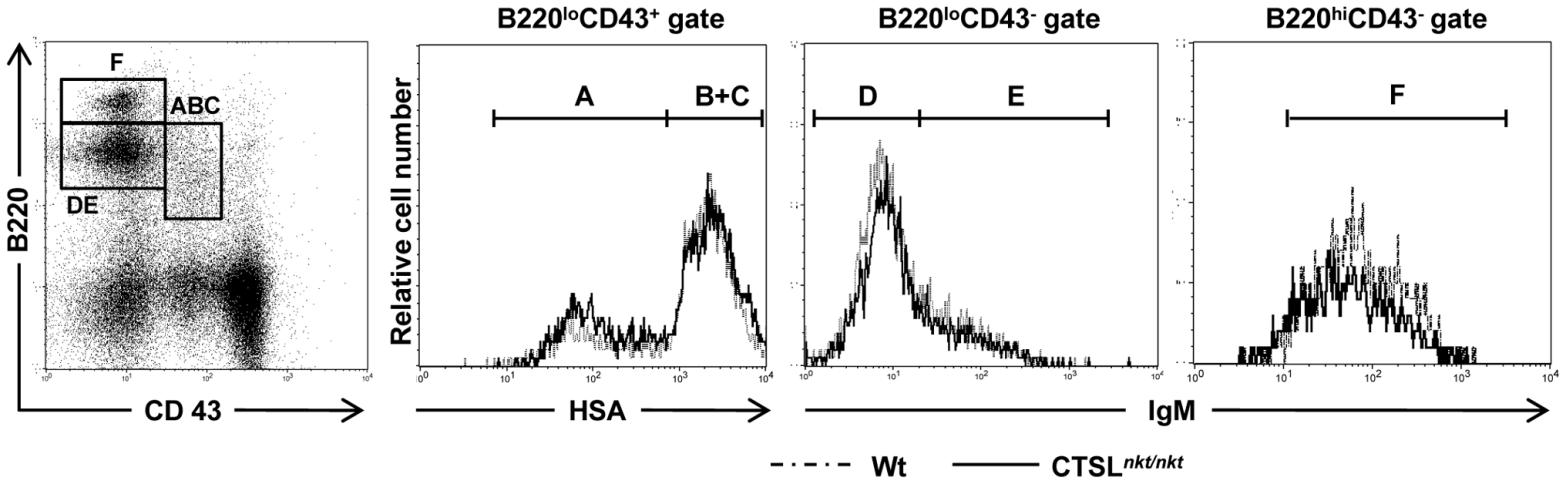
BM B-cell maturation in CTSL*^nkt/nkt^* mice. BM cells from wt and CTSL*^nkt/nkt^* mice were stained with anti-B220, anti-CD43, anti-HSA or anti-IgM. BM B-cell progenitors were classified according to the scheme of Hardy^41^: Fraction A (pre-proB cells: B220^lo^CD43^+^HSA^−/lo^), Fraction B–C (pro-B cells: B220^lo^CD43^+^HSA^hi^), Fraction D (pre-B cells: B220^lo^CD43^−^IgM^−^), Fraction E (immature B cells: B220^lo^CD43^−^IgM^+^) and Fraction F (mature B cells: B220^hi^CD43^−^IgM^+^). Representative results from one out of three independent experiments are shown.

**Figure 5:**
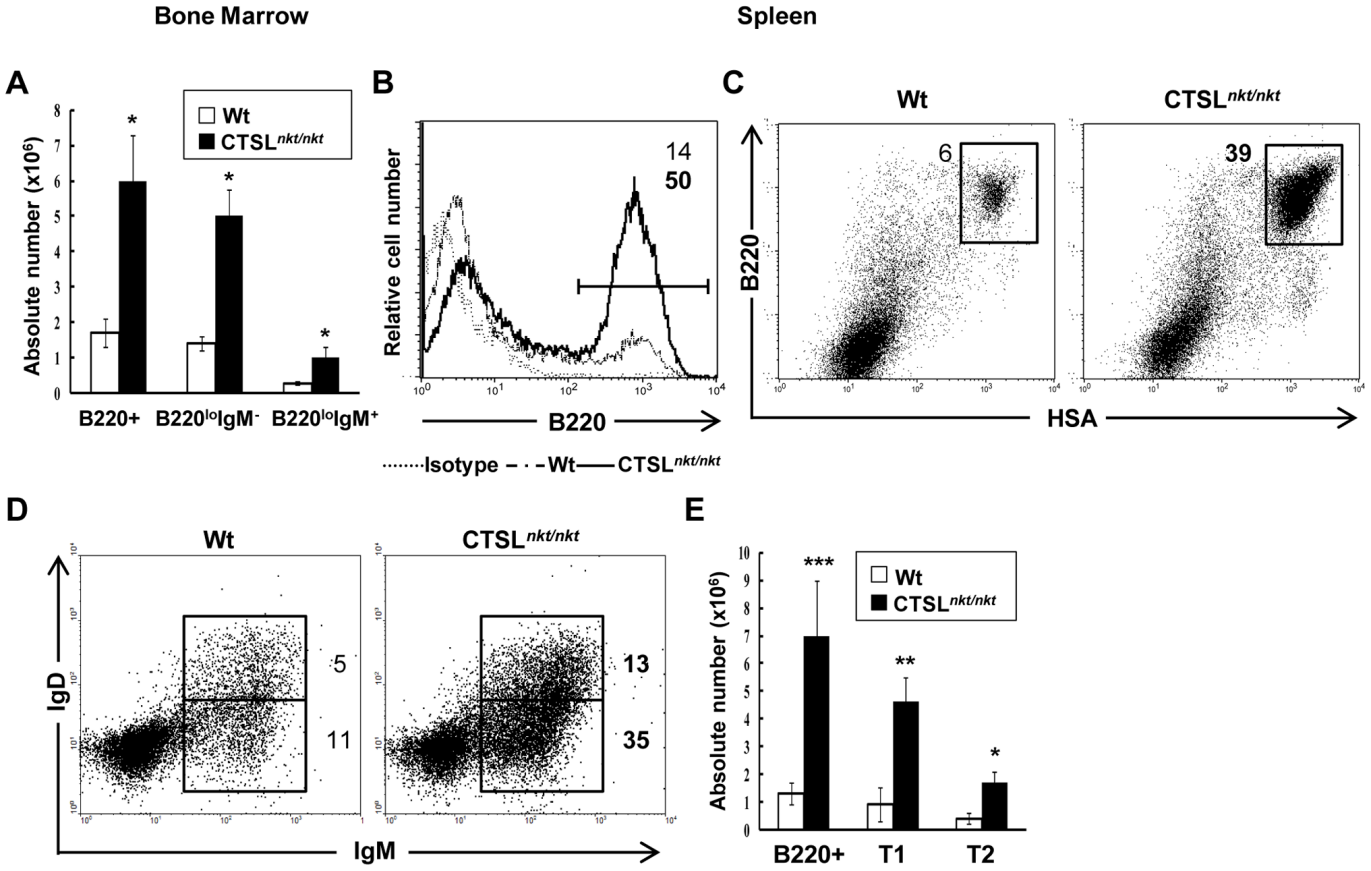
B-lymphopoiesis is increased in CTSL*^nkt/nkt^* mice. Wt and CTSL*^nkt/nkt^* mice received 500 rad of whole-body irradiation. 13 days later, BM and splenic cells were stained with anti-B220, anti-HSA, anti-IgD and anti-IgM before analysis by FACS. (A) Absolute numbers (mean ± SD; n = 9) of BM B220^+^, B220^lo^IgM^−^ and B220^lo^IgM^+^ cells. (B) Representative histograms of splenic B220^+^ cells. (C) Representative dot plots of splenic transitional B cells (HSA^hi^B220^lo/+^). (D) Dot plots of splenic T1 (IgM^hi^IgD^low^) and T2 (IgM^hi^IgD^hi^) B cells. (E) Absolute numbers (mean ± SD; n = 9) of splenic B220^+^, T1 and T2 cells. *p<0.005; **p<0.0005; ***p<0.0001 compared to wt.

We then investigated whether BM B-cell maturation was altered in CTSL*^nkt/nkt^* mice. No differences in total BM cellularity or in total BM leukocyte numbers were detected between normal and mutant mice (Table 1). The percentage and absolute number of pre-pro-B (B220^lo^CD43^+^HSA^−/lo^), pro-B (B220^lo^CD43^+^HSA^hi^), pre-B (B220^lo^CD43^−^IgM^−^), immature (B220^lo^CD43^−^IgM^+^) and mature B cells (B220^hi^CD43^−^IgM^+^) were found to be similar in wt and mutant mice (Fig 4 and Table 1). To investigate *in vivo* BM B-cell production, irradiation-induced autoreconstitution experiments were developed [42]. Mutant and wt mice were sublethally irradiated to eliminate mature and immature lymphocytes. BM stem cells survive this level of irradiation and are able to reconstitute the lymphocyte populations [31]. At day 13, when most of B splenocytes displayed a transitional phenotype (>98% HSA^hi^B220^lo/+^) [43], BM and spleen cells were collected to study B-cell development. As can be observed in Fig 5A, the absolute number of BM B220+ cells was significantly higher (3 fold) in CTSL*^nkt/nkt^* mice. This increase included all BM B-cell developmental stages, except for mature B cells which at this time point were undetectable both in mutant and in wt mice. Regarding the spleen, the percentage and absolute number of transitional B cells (HSA^hi^B220^lo/+^) was about 7 fold higher in CTSL*^nkt/nkt^* mice as compared to wt mice (Fig 5B–E). This increase involved both splenic transitional T1 (IgM^hi^IgD^low^) and T2 (IgM^hi^IgD^hi^) B-cell subsets (Fig 5C and E).

**Table 1:** BM B-cell subsets in wt and CTSL*^nkt/nkt^* mice.

	Absolute number of cells (×10^5^)					
Cell fraction*^a^*	A	B–C	D	E	F	Total BM
Wt	3.9±0.6	11.7±0.6	35.4±3.5	16.5±2.3	10.5±1.7	416.3±19.7
CTSL*^nkt/nkt^*	4.4±0.3	13.0±3.1	36.1±5.5	17.3±4.1	8.9±1.1	418.8±16.5

BM cells from wt and CTSL*^nkt/nkt^* mice were stained with anti-B220, anti-CD43, anti-HSA or anti-IgM mAb before analysis by FACS. Values represent the mean ± SD of cells in each fraction per femur (n = 4). The experiment was performed three times with similar results. *^a^*BM B-cell progenitors were classified according to the scheme of Hardy^41^ as follows: Fraction A (pre-pro-B cells: B220^lo^CD43^+^HSA^−/lo^), Fraction B–C (pro-B cells: B220^lo^CD43^+^HSA^hi^), Fraction D (pre-B cells: B220^lo^ CD43^−^IgM^−^), Fraction E (immature B cells: B220^lo^CD43^−^IgM^+^) and Fraction F (mature B cells: B220^hi^CD43^−^IgM^+^).

These results indicate that CTSL*^nkt/nkt^* mice have an increased BM B cell-production.

### CTSL*^nkt/nkt^* BM SC and precursor B cells lead to increases in the number of CFU-pre–B cells

**Figure 6:**
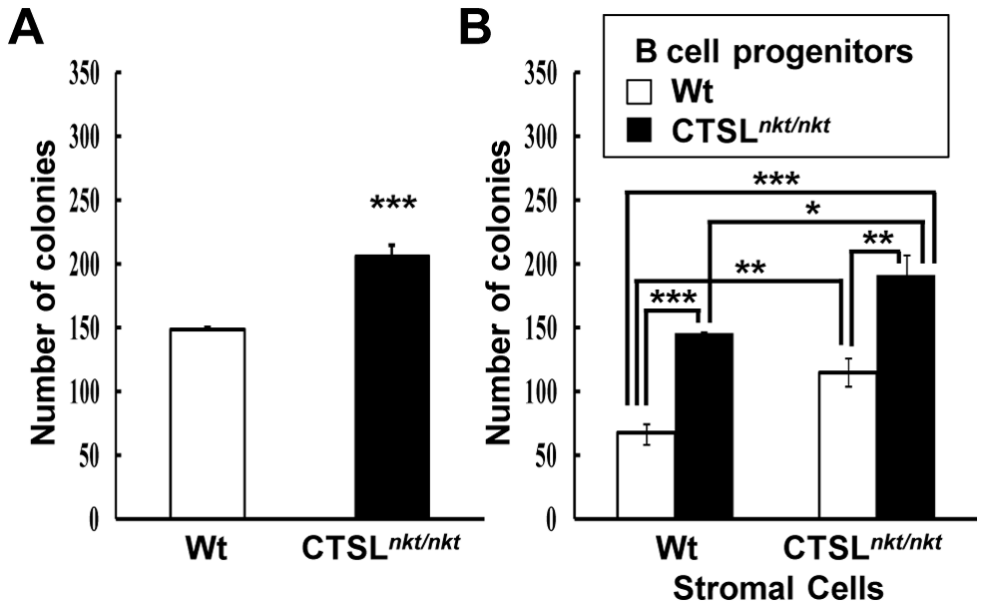
BM SC and precursor cells contribute to the *in vitro* increased B-lymphopoiesis in CTSL*^nkt/nkt^* mice. (A) BM cells from wt and CTSL*^nkt/nkt^* mice were cultured in methylcellulose medium in the presence of rIL-7. Pre-B-cell colonies were enumerated at day 7. (B) Co-culture of pro-B-cell rich populations with BM SC. BM cells from wt and CTSL*^nkt/nkt^* mice were cultured with rIL-7 for 4 days to obtain a population of pro-B cells (B220^lo^CD43^+^HSA^hi^). Pro-B cells rich populations were co-cultured with wt or CTSL*^nkt/nkt^* BM SC monolayers in the presence of rIL-7 for 3 days. Nonadherent cells were harvested, counted, and cloned using the CFU pre-B colony assay system. Data are presented as the mean ± SD of triplicate cultures. *p<0.05, **p<0.005, ***p<0.001 compared to wt.

We first investigated *in vitro* BM B-cell production by performing CFU pre-B assays using day 7 as the end point. Correlating with *in vivo* experiments, a significant increase in the number of pre-B-cell colonies was observed when BM cells from CTSL*^nkt/nkt^* mice were assayed (Fig 6A). To assess the individual contribution of B-cell autonomous and BM microenvironment alterations in causing increases in B-cell production, we investigated the ability of BM SC from wt and CTSL*^nkt/nkt^* mice to support B lymphopoiesis. We first assayed the ability of nonadherent BM cells to form pre-B cell-colonies in the presence of BM SC. Although BM wt cells co-cultured in the presence of CTSL*^nkt/nkt^* BM SC give rise to significantly higher numbers of pre-B colonies than those obtained in the presence of wt BM SC (data not shown), a small number of colonies was obtained even when high numbers of nonadherent BM cells were used. We thus assayed a method described by Tsuboi et al. [32]. Pro-B cell-rich populations from wt or CTSL*^nkt/nkt^* mice were co-cultured with wt or CTSL*^nkt/nkt^* BM SC and the differentiation potential of conditioned progenitor cells into pre-B cells was then assayed. After co-culture with wt SC, the number of pre-B-cell colonies derived from CTSL*^nkt/nkt^* B-cell precursors was 2 fold higher than that derived from wt B cell precursors (Fig 6B), indicating that a B-cell precursor intrinsic alteration affects B lymphopoiesis in CTSL*^nkt/nkt^* mice. Co-cultures between mutant BM SC and wt B-cell precursors also led to a significant increase in B-cell production as compared to co-cultures with wt BM SC. Thus, the lack of CTSL activity on BM SC also contributes to the increased B lymphopoiesis. A further increase in the number of pre-B-cell colonies was observed when CTSL*^nkt/nkt^* B-cell precursors were co-cultured with CTSL*^nkt/nkt^* BM SC, suggesting that the effects of *nkt* mutation on BM SC and B-cell precursors are additive. Overall these results suggest that the increased BM B-cell production observed in CTSL*^nkt/nkt^* mice results from both B-cell autonomous and BM microenvironment features.

### *In vivo* reciprocal BM transplantation experiments indicate that both BM SC and precursor B cells contribute to the increased B lymphopoiesis in CTSL*nkt/nkt* mice

**Figure 7:**
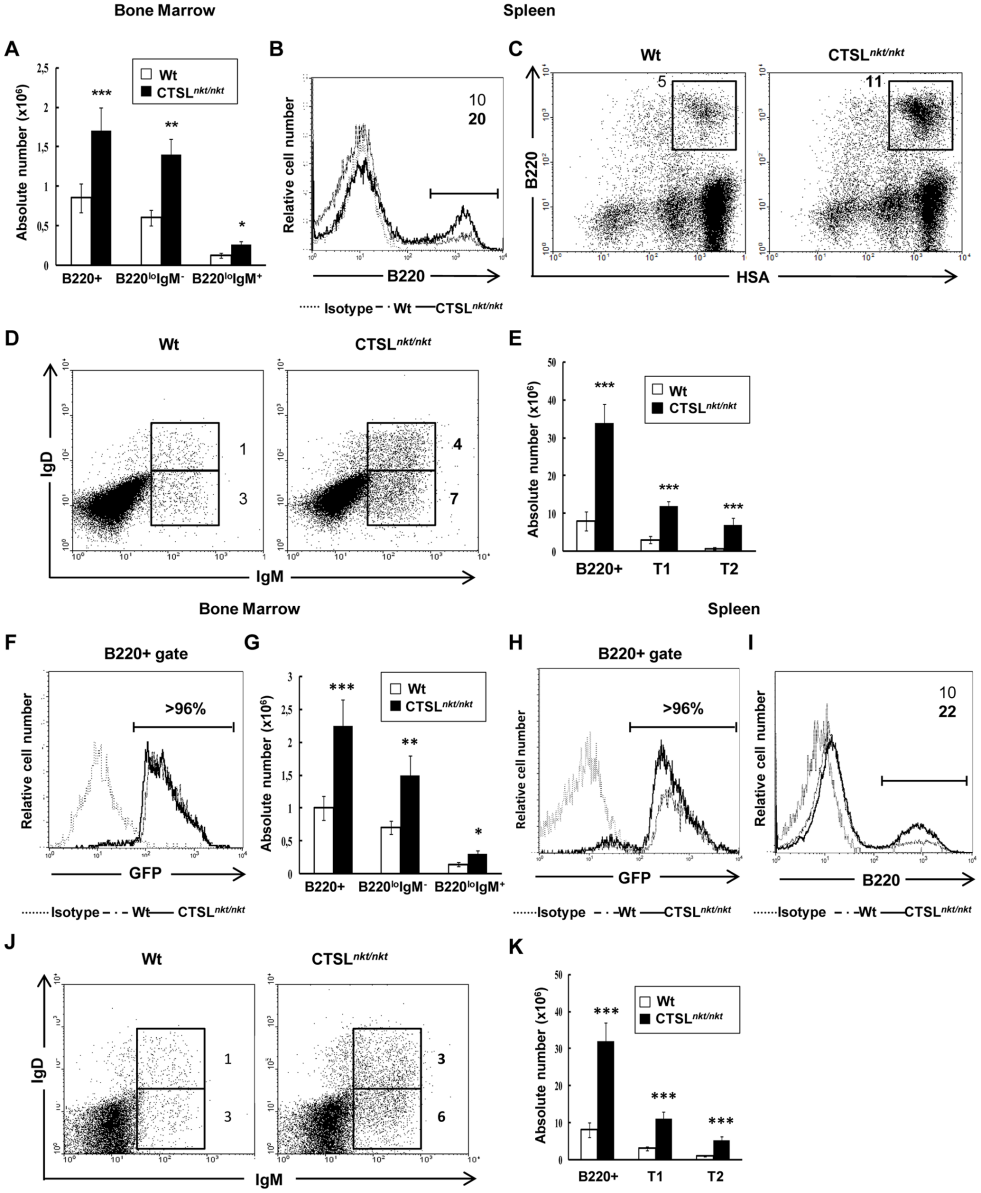
Lack of CTSL activity on BM microenvironment influences the *in vivo* BM B-cell production and the number of transitional B splenocytes. Wt and CTSL*^nkt/nkt^* mice were lethally irradiated and 24 hs later transplanted with wt (A–E) or BALB/c.GFP (F–K) BM cells. Twelve days post-transfer, BM and splenic cells were stained with anti-B220, anti-HSA, anti-IgD and anti-IgM before analysis by FACS. (A) Absolute numbers (mean ± SD; n = 10) of BM B220^+^, B220^lo^IgM^−^ and B220^lo^IgM^+^ cells. (B) Representative histograms of BM B220^+^ cells. (C) Representative dot plots of splenic transitional B cells (HSA^hi^B220^lo/+^). (D) Representative dot plots of splenic T1 (IgM^hi^IgD^low^) and T2 (IgM^hi^IgD^hi^) B cells. (E) Absolute numbers (mean ± SD; n = 10) of splenic B220^+^, T1 and T2 cells. (F) Representative histograms of GFP expression within BM B220^+^ cells. (G) Absolute numbers (mean ± SD; n = 9) of BM B220^+^, B220^lo^IgM^−^ and B220^lo^IgM^+^ cells within GFP+ cells (H) Representative histograms of GFP expression within B220+ splenocytes (I) Representative histograms of splenic B220+ within GFP+ cells. (J) Dot plots of splenic T1 (IgM^hi^IgD^low^) and T2 (IgM^hi^IgD^hi^) cells within GFP+ cells. (K) Absolute numbers (mean ± SD; n = 9) of splenic B220^+^, T1 and T2 cells within GFP cells. *p<0.05; **p<0.005; ***p<0.0005 compared to wt.

In order to evaluate *in vivo* the role of the *nkt* mutation expressed on BM SC, lethally irradiated wt and CTSL*^nkt/nkt^* mice were i.v. inoculated with wt BM cells. In parallel experiments, BALB/c.GFP BM cells were used as donor cells. B220+ (or B220+ GFP+) BM and spleen cells were analyzed twelve days after transplantation by flow cytometry. As can be observed in Fig 7F and H, over 96% of B220+ BM and spleen cells observed in wt and CTSL*^nkt/nkt^* recipients of BALB/c.GPF BM cells were GFP+, indicating that the reconstituting B cells were derived from GFP+ donor BM cells. As can be observed in Fig 7A and G, CTSL*^nkt/nkt^* recipients of wt or BALB/c.GFP+ cells showed higher absolute numbers of precursor-B cells than wt recipients. Regarding the spleen, both the percentage and absolute numbers of transitional B-cells were higher in CTSL*^nkt/nkt^* recipients (Fig. 7B–E and I–K). Two mo after transplantation B220^+^ transitional splenocytes remained increased in CTSL*^nkt/nkt^* recipients (Data not shown). These results indicate that the absence of CTSL activity on BM microenvironment influences BM B-cell production and the number of transitional B splenocytes.

**Figure 8:**
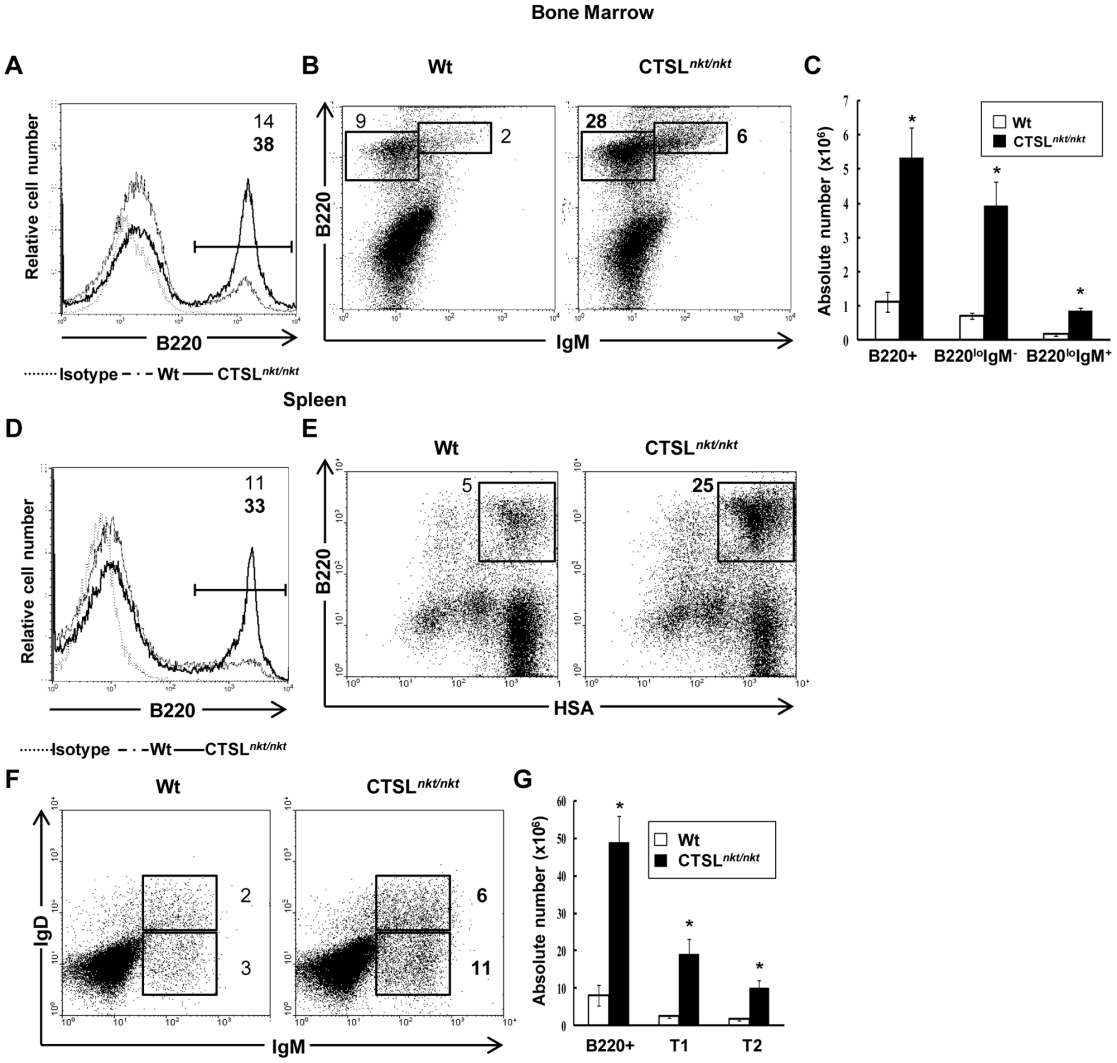
The lack of CTSL activity affects the intrinsic ability of BM progenitor cells to develop into B cells. Wt mice were lethally irradiated and 24 hs later transplanted with wt or CTSL*^nkt/nkt^* BM cells. Twelve days later, BM and splenic cells were stained with anti-B220, anti-HSA, anti-IgD and anti-IgM before analysis by FACS. (A) Representative histograms of BM B220^+^ cells. (B) Dot plots of BM B-cell precursors (B220^lo^IgM^−^ and B220^lo^IgM^+^ cells). (C) Absolute numbers (mean ± SD; n = 10) of BM B220^+^, B220^lo^IgM^−^ and B220^lo^IgM^+^ cells. (D) Representative histograms of splenic B220^+^ cells. (E) Representative dot plots of splenic transitional B cells (HSA^hi^B220^lo/+^). (F) Representative dot plots of splenic T1 (IgM^hi^IgD^low^) and T2 (IgM^hi^IgD^hi^) cells (g) Absolute numbers (mean ± SD; n = 10) of splenic B220^+^, T1 and T2 cells. *p<0.0005 compared to wt.

To investigate whether the *nkt* mutation on BM hematopoietic progenitor-cells is also able to influence *in vivo* B cell lymphopoiesis, BM cells from wt or CTSL*^nkt/nkt^* mice were transferred into lethally irradiated wt mice. As can be observed in Fig 8A–C, wt recipients that received CTSL*^nkt/nkt^* BM cells had higher percentages and absolute numbers of BM B-cell precursors than those that received wt BM cells. These mice also showed increased percentage and absolute numbers of transitional (T1 and T2) splenic cells (Fig 8D–G). These results indicate that a B-cell precursor intrinsic alteration also plays a role in the increase in BM B-cell production and in the increase in transitional B-cell numbers observed in CTSL*^nkt/nkt^* mice.

Our *in vitro* and *in vivo* results indicate that CTSL activity both in BM SC and in hematopoietic progenitor cells is able to negatively regulate BM B cell production and the number of transitional B splenocytes.

## Discussion

We have previously shown that the *nkt* mutation broadly affects the maturation and the homeostasis of T-cell populations. Results presented herein show that in CTSL*^nkt/nkt^* mice BM B-cell production and emigration are increased, leading to increases in peripheral B-cell numbers. In the enlarged LN from mutant mice, the number of B cells was found to be highly increased. LN B cells were mature according to their expression of surface markers. In addition, we have previously shown that CTSL*^nkt/nkt^* mice produce specific IgG against OVA [34], indicating that B cells of mutant mice are functional.

The fact that LN B cells did not show alterations in basal apoptosis and proliferative levels suggest that a higher input of B cells into LN could be involved in the increased number of LN B cells.

It is well-known that ECM components influence the homeostasis of lymphocytes in lymphoid organs [43]–[48]. We have previously demonstrated that several ECM components are increased in the LN of CTSL*^nkt/nkt^* mice [25].Thus, it can be hypothesized that increases in LN ECM components may increase the number of niches for B (and T) lymphocytes. Therefore increased numbers of B cells could not only enter but survive in the altered environment of CTSL*^nkt/nkt^* LN. In support of this hypothesis, the absence of alterations in ECM components in the spleen of CTSL*^nkt/nkt^* mice [25] correlates with the presence of only slightly increased numbers of splenic B cells.

Regarding the spleen, normal numbers of FM B cells and increased numbers of transitional T1, T2 and the minor subset of MZ cells were observed.

Regarding the BM, a clear increase in CTSL*^nkt/nkt^* BM B-cell production was detected both *in vitro* (CFU-pre-B assays) and *in vivo* (autoreconstitution experiments). Of interest, no alterations in the total number of B-cell precursors were detected. In addition, their proliferative or apoptosis levels were neither altered (data not shown). Based on BrdU pulse-chase experiments, we could demonstrate that higher numbers of immature B cells leave the BM and reach the spleen of CTSL mutant mice.

Collectively, our data show that both BM B-cell production and emigration are increased in CTSL*^nkt/nkt^* mice leading to an increase in the number of peripheral B cells, notably in follicular LN B-cells.

It is known that BM SC regulates BM B-cell development through the secretion of various cytokines, growth factors and hormones that affect progenitor cell growth, differentiation, and/or survival [49], [50]. Contact of B-cell progenitors with SC and ECM proteins is also involved in BM B-cell development [51]. Alterations in the intrinsic ability of BM progenitor cells to develop into B cells could also affect BM B-cell production. Our *in vitro* data led us to hypothesize that the nkt mutation could influence B-cell production by acting both on BM SC and BM B-cell precursors. To confirm this hypothesis, *in vivo* reciprocal BM transplantation experiments were performed. Our results showed that CTSLnkt/nkt hosts receiving wt or GFP.BALB/c cells had higher numbers of BM B-cell precursor and transitional B-splenocytes as compared to wt hosts, thus confirming that the nkt mutation influences B-cell production by altering the BM SC. Besides, the lack of CTSL activity would also affect the intrinsic ability of BM progenitors to develop into B cells, as indicated by the increase in the number of both BM B-cell precursors and transitional B splenocytes in wt mice transplanted with BM mutant cells.

Taken together, these findings point both to alterations in BM SC and in hematopoietic progenitor cells as contributing to the increased B-cell hematopoiesis in mutant mice.

The molecular mechanisms that underlie the increased BM B-cell production in CTSL*^nkt/nkt^* mice remain to be defined. To date, CTSL activity on factors known to be involved in B-cell hematopoiesis has only been studied in tissues and organs other than BM. Of note, it has been reported that CTSL-deficient skin fibroblasts show a decreased degradation of insulin-like growth factor binding protein-3 (IGFBP-3) from insulin-like growth factor-1 (IGF)/IGFBP complexes [52]. Limited proteolysis of IGFBP from IGF/IGFBP complexes constitutes a central mechanism by which complexed IGFs are released from IGFBPs increasing IGF bioavailability [53]. Considering that IGF-1 stimulates BM B-cell production [54], [55], it seems unlikely that a putative decrease in IGF-1 release in the BM of CTSL*^nkt/nkt^* mice would be directly involved in the increases registered in BM B-cell production. Moreover, decreased degradation of IGFBP-3 from IGF/IGFBP complexes in CTSL-deficient fibroblasts also results in an extracellular accumulation of IGFBP-3 [52]. Since IGFBP-3 inhibits human pro-B-cell development *in vitro* [56], these data do not support the hypothesis that a putative decrease in IGFBP-3 proteolysis could account for the increased BM B-cell production of CTSL*^nkt/nkt^* mice.

In a model of myocardial infarction, it has been shown that the expression of stem cell factor and stromal cell-derived factor-1 were significantly decreased in the myocardium of CTSL KO mice [57]. Since both factors show positive effects on B-cell hematopoiesis [58], [59], a putative similar effect of the lack of CTSL in BM would not play a direct role in the increases in B-cell hematopoiesis reported herein.

On the other hand, it has also been recently shown that CTSL is able to block AKT signaling in cardiomyocytes [12] and keratinocytes [17]. Studies on PI3K/AKT axis in B lymphocytes have established the importance of this pathway in affecting B-cell proliferation, differentiation and associated molecular events such as V(D)J recombination and class switch recombination [60]. Besides, it has been shown that AKT activation in BM endothelial cells up-regulates specific angiocrine factors that support the expansion of hematopoietic stem and progenitor cells, whereas activation of both AKT and MAPK favors maintenance and lineage-specific differentiation of these cells [61]. It cannot be discarded that an increased activation of the AKT pathway in CTSL*^nkt/nkt^* B-cell precursors and/or BM endothelial cells could be involved in the increased BM B-cell hematopoiesis.

**Figure 9:**
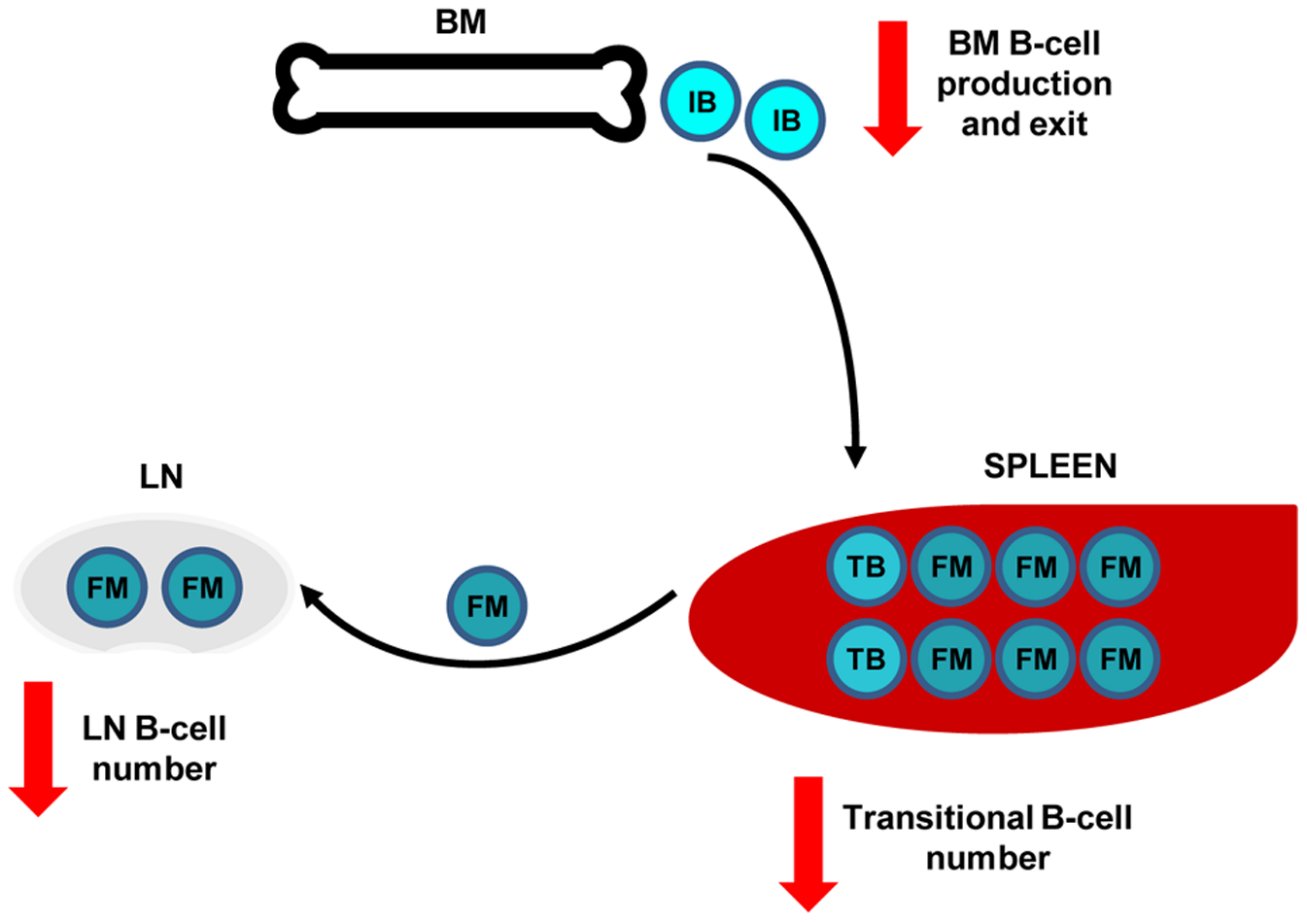
Influence of CTSL on B-cell homeostasis. By acting both on BM B-cell precursors and SC, negatively regulates the BM production and the export of immature B cells to the periphery. As a consequence, the number of blood and spleen TB and LN FM B cells is restricted by CTSL activity. Besides, by affecting the levels of expression of LN ECM components, CTSL would also limit the number of LN niches for FM B cells, cooperating in that way to regulate the number of FM B cells . IB: immature B cells; TB: transitional B cells; FM: follicular mature B cells.

Even though there is accumulating evidence for specific functions of CTSL in health and disease, to our knowledge this is the first report showing that CTSL plays an important role in the regulation of the homeostasis of the B-cell pool. As schematized in Fig 9, by acting both on BM hematopoietic progenitor cells and SC, CTSL negatively regulates BM B-cell production. Besides BM immature B-cell emigration is also restrained by CTSL activity. As a consequence, CTSL negatively regulates the number of spleen and blood transitional B cells and LN FM B cells. Besides, by affecting the levels of expression of LN ECM components, CTSL would also limit the number of LN niches for FM B cells, cooperating in that way to regulate the number of FM B cells.
